# Homogeneous Distribution of Polymerizable Coumarin Dyes for Active Few Mode POF †

**DOI:** 10.3390/ma13081975

**Published:** 2020-04-23

**Authors:** Florian Jakobs, Kristoffer Harms, Jana Kielhorn, Daniel Zaremba, Pen Yiao Ang, Wolfgang Kowalsky, Hans-Hermann Johannes

**Affiliations:** Institut für Hochfrequenztechnik, Technische Universität Braunschweig, 38106 Braunschweig, Germany; florian.jakobs@ihf.tu-bs.de (F.J.); kristoffer.harms@ihf.tu-bs.de (K.H.); jana.kielhorn@ihf.tu-bs.de (J.K.); daniel.zaremba@ihf.tu-bs.de (D.Z.); pen.yiao.ang@ihf.tu-bs.de (P.Y.A.); wolfgang.kowalsky@ihf.tu-bs.de (W.K.)

**Keywords:** dye doped fiber, analysis method, dye distribution, copolymerization, few mode

## Abstract

For most kinds of active polymer optical fibers, a homogeneous distribution of dye molecules over the entire fiber length and cross section is required. In this study, chemical bonding of dyes to *poly*(methyl methacrylate) (PMMA) by copolymerization is achieved within the polymerization process instead of dissolving the dyes in the monomers. In combination with an improved fabrication mechanism, this leads to homogeneous dye distribution within the preforms. A method for proving the integration of the dyes into the polymer chains has been developed using high-performance liquid chromatography (HPLC) and size exclusion chromatography (SEC). Prestructured core-cladding preforms with dye-doped *poly*(cylohexyl methacrylate-*co*-methyl methacrylate)-core have been prepared with the Teflon string technique and were heat-drawn to few mode fibers.

## 1. Introduction

Using highly efficient luminescent dyes in optical *poly*(methyl methacrylate) (PMMA) matrices is a key step in the development of emerging technologies such as polymer fiber-based solar collectors (FFSCs), sensors, contactless coupling devices, and full-polymer fiber lasers [1]. For all these applications, a homogeneous distribution of the dye molecules within the polymer matrix—also in preforms and optical fibers—is required to avoid quenching effects due to dye agglomerations [2]. To achieve the best homogeneous distribution, a statistical MMA-dye-copolymer is used. Different kinds of polymerizable dyes have been shown in previous works [3,4,5,6,7]. However, it has not been proved that dye molecules and polymers formed copolymers.

To demonstrate that our polymerizable dyes not only act in a host–guest system as incorporated dopants in this publication, we show a systematically study and an experimental method to verify the formation of dye–polymer chains. The study consists of three different examinations based on optical and chromatographic analytical methods: ultraviolet–visible (UV/VIS) spectroscopy spectroscpopy, high-performance liquid chromatography (HPLC), and size exclusion chromatography (SEC). Furthermore, the dye distribution in preforms was examined and improved by the use of polymerizable dyes in combination with an adapted preform process. From this information, we were able to produce dye-doped few-mode optical polymer fibers. For all the tests, two coumarine dyes with the same chromophore were used, as these molecules have quite simple structures with low molecular weight and they show numerous possibilities from chemical modification to tailor-made.

## 2. Materials and Methods

### 2.1. Chemical Reagents and Materials

Solvents, cyclohexyl methacrylate (CHMA), and lauroyl peroxide (97.0%) were purchased from Sigma-Aldrich (St. Louis, MO, USA). *n*-Butyl-mercaptan (99.0%) was purchased from Acros Organics (Morris Plains, NJ, USA). Methyl methacrylate (MMA) was obtained from Tokyo Chemical Industry Co., Ltd. (Tokyo, Japan). Monomers and technical-grade solvents were distilled prior to use. All other chemicals were used without further purification.

### 2.2. Dyes

**Figure 1:**
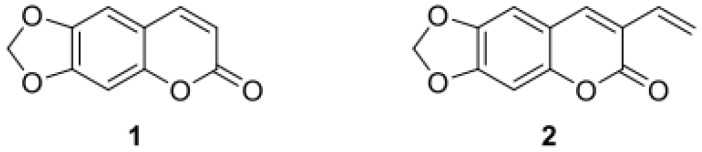
Ayapin dye **1** without terminal vinyl group (**left**). 3-Vinylayapin dye **2** with terminal vinyl group (**right**).

Two coumarin dyes, 6,7-(methylenedioxy)-coumarine (ayapin **1**) and 6,7-(methylenedioxy)- 3-vinylcoumarine (3-vinylayapin **2**), with a similar chromophore have been synthesized as shown in Section 2.3. The structural formulas of the dyes are shown in Figure 1. 3-Vinylayapin has a terminal vinyl group that allows the linkage of the dye into the polymer chain. In this work, 3-vinylayapin is referred to as the polymerizable dye, and ayapin is identified as the non-polymerizable dye.

### 2.3. Synthesis of 6,7-(Methylenedioxy)-3-vinylcoumarine

**Figure 2:**
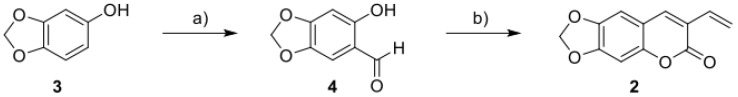
Reaction conditions (a) sesamol (**3**) (1 eq.), tin(IV) chloride (1.2 eq.), dichloromethoxymethane (1 eq.), dichloromethane (DCM), 0 °C to room temperature in 3 h, acid workup with 1 m HCl, 83% (b) **4** (1 eq.), 3-butenoic acid (1.25 eq.), N,N-Dimethylpyridin-4-amine (1.25 eq.), dicyclohexylmethanediimine (1.25 eq.), Cs_2_CO_3_ (1 eq.), DCM, 48 h, 43%.

For the synthesis of 3-vinylayapin (**2**), the synthetic pathway shown in Figure 2 was followed. The first step is a formylation of commercial available sesamol (**3**) according to a procedure published by RIECHE [8]. The resulting aldehyde **4** was converted in a one-pot synthesis with 3-butenoic acid to 3-vinylayapin. Dye **2** is a known substance and a detailed mechanism of the reaction can be found in the literature [9].

### 2.4. Sample Preparation

Distilled methyl methacrylate (MMA) was mixed with lauroyl peroxide and dye molecules in different concentrations between 0.0005 mol% and 0.3 mol%. The mixture was saturated with nitrogen and *n*-butyl-mercaptan was added. Afterwards, the solution was filtered through a 0.45 μm polytetrafluoroethylene (PTFE) syringe filter into screw cap glass vials and polymerized at 60 °C for 24 h and 100 °C for 48 h. After the polymerization process, the glass vials were removed and the polymer bulk samples were cleaned with isopropanol and nitrogen.

In the established bulk polymerization preform process (“Process A”), the same monomer solution is filtered into sample tubes and then polymerized for five days at a linear rising temperature. The final temperature of 100 °C was held for 24 h before samples were cooled to room temperature for another 24 h. In an alternative preform process (“Process B”), an orbital shaker is used. Polymerization is performed at 250 rpm at discrete temperature steps at 60° for 48 h and another 105° for 24 h. The cleaning step for both preform processes is similar to bulk sample cleaning.

**Figure 3:**
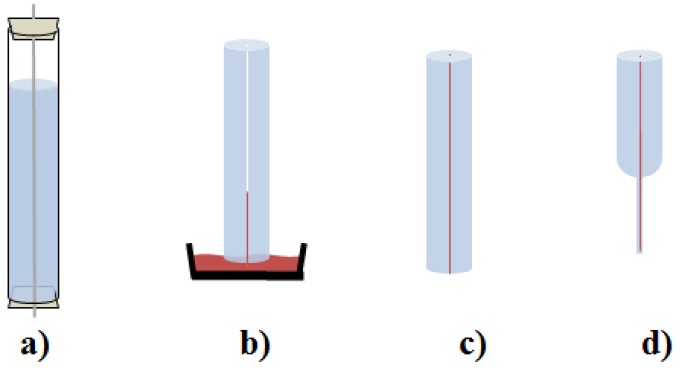
Schematic of core–shell preform production. (**a**) Fabrication of hollow-core PMMA preform. (**b**) Filling in the doped CHMA:MMA core material. (**c**) Polymerization process. (**d**) Near-single mode (SM) fiber is achieved by fiber drawing process.

For the core–shell preforms, a two-step process was used. In the first step, a hollow core preform is produced. Therefore, cleaned glass tubes are prepared with an teflon-coated stainless steel wire as a space-holder for the core. Undoped monomer solution is filled in and the tubes were sealed (Figure 3a). The wires are put under tension to prevent deformation during polymerization. For polymerization, process A is used. After removing the glass tubes and wires, the ends of the preforms are cut of and the end faces are polished roughly. In an second step, a mixture of MMA and CHMA is treated as described for the pure MMA. The mixture is filled into the cavity of the prepared preform and the ends were sealed (Figure 3b). Temperature process A is performed with the filled preform to achieve a core–shell preform (Figure 3c). The basic technique has already been described in literature [10,11]. Polymer optical fibers with small core diameters were drawn from the preforms by a fiber drawing process (Figure 3d).

For SEC analysis, the polymer bulk samples were dissolved in tetrahydrofurane (THF) at a concentration 1 mg·${\mathrm{mL}}^{-1}$. The solution was filtered through 0.45 μm polyethylene terephthalate (PET) syringe filters before analyzation. For HPLC measurements, the polymer was just dissolved in THF. Afterwards, the solution was precipitated with methanol and the precipitate is removed. The residual solvent was dried by using a vacuum process and then resolved in acetonitrile at a defined ratio. For photoluminescence (PL) spectroscopy and fluorescence lifetime measurements polymer bulk samples and preforms were cut into discs. Samples were polished roughly. For UV–Vis spectrometry, sample cuts from the preforms were dissolved in dichloromethane (DCM) with a ratio of 0.1 g polymer in 10 mL of DCM.

### 2.5. Methods

HPLC analysis has been performed for resolved residuals in acetonitrile (ACN) of the precipitated polymer bulk samples. The polarity selective HPLC is used in reversed phase mode. The set-up uses C_8_-bonded silica columns with a length of 250 mm, a diameter of 4.6 mm, and a particle size of 5 μm. It offers a hydrophobic stationary phase; consequently, less polar molecules will pass slower than polar compounds [8]. An isocratic method with H_2_O/ACN (1:1) at a flow rate of 1 mL·min^−1^ and 22 °C column temperature is used.

Solved polymer bulk samples have been analyzed by size exclusion chromatography (SEC). SEC analysis was performed on a modified Agilent Technologies SECcurity GPC 1260 Infinity System (Agilent Technologies, Inc., Santa Clara, CA, USA) with autosampler and WinGPC UniChrom software V8.00 from PSS (Mainz, Germany). The system was calibrated with a PSS ReadyCal-Kit for PMMA M_*p*_ 800 to 182,000 g·mol^−1^ and polystyrene $M_{p}$ 682 to 2,520,000 g·mol^−1^. As concentration-selective detectors, Agilent 1260 RID RI-detector and Knauer WellChrom Spectro-Photometer K-2501 UV detector (Knauer Wissenschaftliche Geräte GmbH, Berlin, Deutschland) were used. As molar mass-sensitive detector, PSS viscosity detector PSS Security DVD 1260 was used. As separation columns, PSS SDV 5 μm 1000 Å, 100,000 Å and 1,000,000 Å columns were used. The measurements were performed in HPCL grade THF with injection volume of 50 μL and a flow rate of 1000 mL·min^−1^ at 35 °C.

## 3. Results

### 3.1. Proof of Copolymerization

**Figure 4:**
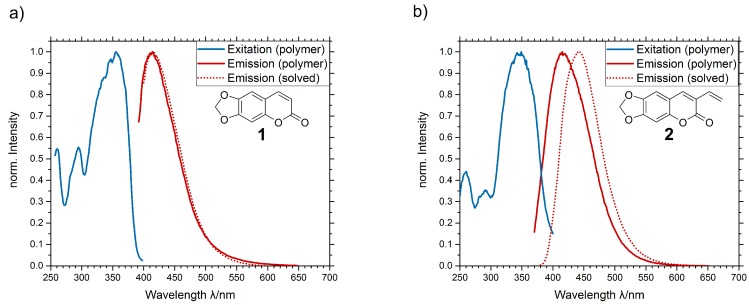
Photoluminescence spectra of (**a**) ayapin and (**b**) 3-vinylayapin.

The optical properties of the two dye molecules show individual behavior in different environment. PL spectra have been gathered from both dye molecules solved in THF as well as in bulk PMMA, prepared as described in Section 2.4. Results are shown in Figure 4. No significant spectral shift can be detected for in the unpolymerizable ayapin dye **1** between the solved sample and the polymer sample. The peak emission wavelength is located at 412 nm for the solved dye and 414 nm for the dye in polymer. The small peak shift is caused by different polarity of the solvent. Compared to the unpolymerizable dye **1**, the solved 3-vinylayapin **2** has a red-shifted emission peak of 442 nm due to the terminal vinyl group. It acts as an donor which pushes electrons into the chromophor causing the red shift. In the PMMA matrix, the 3-vinylayapin does not show this effect. The emission spectrum peak is located at 413 nm, which is the same as the emission peaks of the unpolymerizable dye **1**. The vanishing shift of the vinyl dye **2** is caused by a reaction of the terminal vinyl group with the polymer matrix during polymerization.

**Figure 5:**
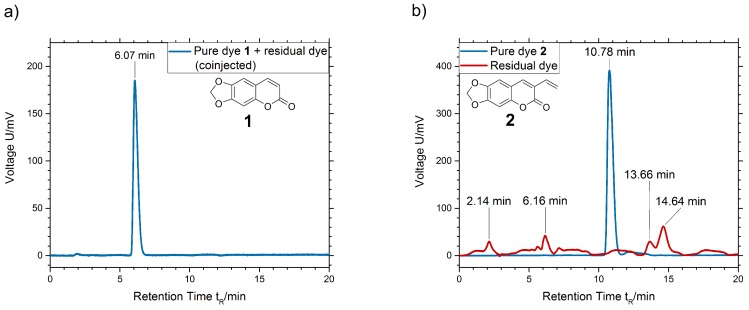
High-performance liquid chromatography (HPLC) chromatogram of (**a**) ayapin **1** and (**b**) pure dyes and residuals from precipitation for 3-vinylayapin **2**. As reference, both dyes **1** and **2** were used in concentrations of 0.001 mol·$L^{-1}$.

Comparing the data from HPLC analysis, we get evidence that a reaction at the terminal vinyl group of dye **2** takes place. From solving and precipitating the polymer samples, we gain the polymer precipitate, on the one hand, and the solvent-optionally containing residuals on the other hand. Residuals may be dye molecules that are not connected to the polymer chains: pure ayapin **1** or oligomers of 3-vinylayapin **2**. The polymerizable 3-vinylayapin molecules, which are supposed to be connected to the polymer chains, will stay in the precipitated polymer. Pure ayapin and residuals have been injected together (Figure 5a). The coinjection shows a smooth baseline with a single peak at a retention time of 6.07 min. This indicates no reaction of ayapin **1** during the polymerization process.

In contrast, the chromatogram of 3-vinylayapin (Figure 5b) shows multiple weak peaks for the residual dye (red line) at retention times of 2.14, 6.16, 13.66, and 14.64 min. For those peaks, several explanations are possible, especially if one takes the free radical polymerization mechanism into account. Due to the scope of our paper the formation of oligomers, formed by the 3-vinylayapin, seems reasonable. All peaks show absorption peaks between 340 and 360 nm, which is the characteristic of the basic coumarine structure. Further reactions between the dyes molecules, e.g., the formation of dimers, oxidation products, or bridged species, could likely have expired. At this point, real structural clarification goes beyond the scope of the presented work. Nevertheless, according to the experimental HPLC set-up, the unpolar species eluting at 13.66 and 14.64, and thus later than the reference sample, might belong to formed oligomers, whereas the peaks at 2.14 and 6.16 belong to unidentified dye species of higher polarity. Retention times are characteristic for the existence of a certain substance. It is significant that there is no peak at the retention time 10.78 min of the pure dye **2** (blue line). Thus, there is no pure 3-vinylayapin left in the residual, but traces can be found in the precipitated polymer fallout.

Similar results were achieved by size exclusion chromatography (SEC). In this method, the time that the samples need to travel through the columns depends on their size: smaller molecules stay longer in the column than bigger ones. In this work, SEC is used to analyze doped polymer samples. Small dye molecules will take longer to pass the column than the lager polymer chains. The method provides information on whether the dye molecules are connected to the polymer chains.

**Figure 6:**
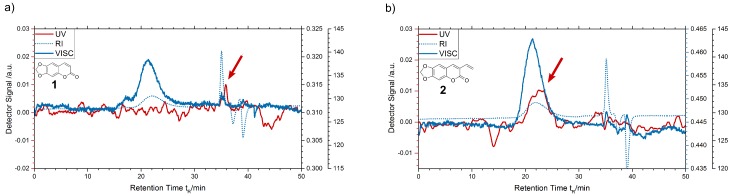
Size exclusion chromatography (SEC) chromatogram of (**a**) ayapin **1**-doped polymer and (**b**) 3-vinylayapin **2**-doped polymer. The blue lines are detector signals of refractive index (dotted) and viscosity detectors (solid), the red lines represent the signal of UV detector.

For the measurements, three different detectors were used: refractive index (RI), viscosity (VISC), and UV-absorption (UV) detectors. The RI and VISC detector are mainly used to gather information about molecular weight ($M_{n}$ and $M_{w}$), molecular mass distribution, and dispersity (Ð) of the polymer. The UV detector is used at wavelength of 355 nm, which is the peak absorption wavelength of the dye molecules. Thus, the detector only responds to the dye molecules and not the the PMMA, which absorbs at wavelength below 300 nm. It should be noted that the the significance of the arrow-pointed peak is quite low, as the UV-detector is operating just slightly above the noise floor due to the small amounts of dye. Both polymer samples, unaffected by their dotation, show peaks of RI and VISC detectors at the same retention times. The results from polymer doped with ayapin **1** show that the small, unreacted dye molecules will take much longer than the polymer chains to reach the detector. They are flushed out with the waste at 35 to 40 min retention time (see Figure 6a). A details analysis is not reasonable here as 44 min signal is detected at the edge of the separating capacity of the SEC column (which can be seen at the RI and VISC detector as well). The signals at 36 and 44 min retention time both belong to the dye and are not bonded to the matrix are dissolved to the THF with off-average molecular weight host polymer. In contrast, the polymer doped with 3-vinylayapin **2** shows an overlap of the polymer and the dye signal. The dye molecules reach the detector simultaneously to the polymer at 20 to 25 min retention time (Figure 6b). This proves that the 3-vinylayapin molecules have successfully been integrated into the polymer chains. Furthermore, no significant change of the number averaged molecular weight ($M_{n,3-vinylayapin}$ = 1.05 × $10^{5}$ g·${\mathrm{mol}}^{-1}$) or dispersity (${Ð}_{3-vinylayapin}$ = 1.81) in comparison to undoped ($M_{n,PMMA}$ = 1.08 × $10^{5}$ g·${\mathrm{mol}}^{-1}$, ${Ð}_{PMMA}$ = 1.67) or ayapin-doped PMMA ($M_{n,ayapin}$ = 1.03 × $10^{5}$ g·${\mathrm{mol}}^{-1}$, ${Ð}_{ayapin}$ = 1.87) is observed. This indicates no reaction of 3-vinylayapin with initiator molecules or chain transfer agents.

### 3.2. Dye Distribution within the Preform

A homogenous dye distribution in the preforms is a key factor in the processing of active polymer optical fibers. Depending on the solubility of the dye in the monomers and the preform production process, a dye concentration gradient may occur.

The polymerization in upright standing glass tubes does not run off uniformly in the whole preform at the same time.

In this study, the dye concentration gradient of preforms could be reduced significantly by the use of polymerizable dyes in combination with an improved prefrom fabrication technique. To qualify the amount of dye in the preform, the doped polymer is solved in dichloromethane (DCM) at a defined ratio. Samples are analysed with UV–Vis spectroscopy. This defined ratio allows a comparison of the different absorption measurements in order to compare the amount of dye molecules directly. As there is no calibrated reference with an certain amount of dye, no absolute numbers for the dye concentrations can be given.

Dye-doped preforms have been fabricated with both processes described in Section 2.4. Process B, which uses an orbital shaker, is also able to reduce the TROMMSDORFF–NORRISH effect [12] by improved mixing, and therefore creating a balanced viscosity to avoid high viscosity hotspots (It should be noted that no TROMMSDORFF–NORRISH effect was observed when using the oven process (A). The temperature slope in that process has been in optimized in order to avoid gel effect. In contrast, we observed gel effect issues when using discrete temperature steps as in process B without the use of an orbital shaker or other stirring mechanism). Preforms doped with ayapin **1** and 3-vinylayapin **2** have been produced with process A and B. From all four preforms, samples from top and bottom have been solved and analyzed. The gap between the absorption peaks of the top and bottom sample is the value for the dye concentration gradient. Table 1 gives an overview over the samples.

**Figure 7:**
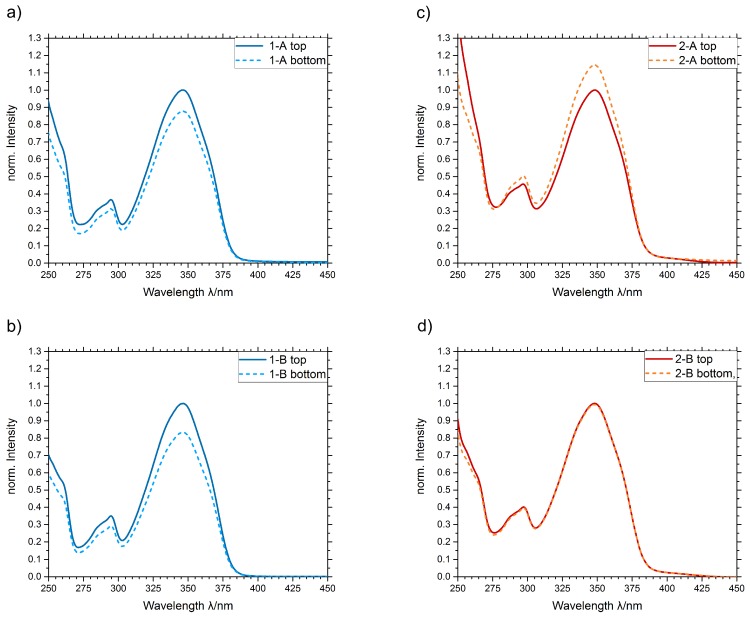
Absorption spectra of the produced preforms. Solid lines represent the samples from the top, dashed lines represent the samples from the bottom of the preforms. (**a**) Sample 1-A. (**b**) Sample 2-A. (**c**) Sample 1-B. (**d**) Sample 2-B.

The absorption spectra in Figure 7 show differences in the top and bottom dye concentrations for samples 1-A, 2-A, and 1-B. The polymerization starts at the outside and at the bottom of the preform. The polymer is growing from outside to inside and from bottom to top. Due to the increasing viscosity of the monomer mixture, the solubility of the unpolymerizable vinylayapin dyes **1** in the mixture decreases. The dye molecules that cannot be solved anymore tend do gather in upper area of the preforms where the viscosity is still lower, and therefore the solubility is higher. As the viscosity rises with ongoing polymerization, the dye molecules get immobilized at the top area thus the dye concentration is higher. In contrast, the polymerizable 3-vinylayapin dye **2** shows an opposite effect. The dye concentration is higher at the bottom part. Due to the nonuniform polymerization, the dye molecules get integrated in the growing polymer chains at the bottom of the preforms. As the overall dye concentration lowers, the molecules are not affected as strong by the viscosity-dependent solutability drop as the unpolymerizable dye molecules. Therefor, the vice versa effect can be observed for the polymerizable dye molecules.

The combination of polymerizable dyes and the orbital shaker process is able to reduce the concentration gradient within the preform to zero (Figure 7c). The growing polymer chains with the integrated dye molecules are distributed homogeneously in the preform by the motions of the shaker. This allows to produce homogeneous doped preforms by bulk polymerization.

### 3.3. Active Few-Mode Fibers

To achieve waveguiding, materials with two different reflective indices *n* are required. In a basic step index optical fiber, the guiding fiber core with higher *n* is surrounded by an cladding with lower *n*. For producing this structure, different options are available, such as coextrusion or a two-step process in which a core fiber is drawn and cladded afterwards. Single or few mode fibers requires core diameters $d_{K}$ in the range of a few micrometers, depending on the refractive index difference (expressed as the numerical aperture $A_{N}$) between core and cladding and the wavelength λ of the guided light. The *V* number (Equation (1) is used in this context. For *V* numbers below 2.405, only one mode (per polarization direction) can propagate in the fiber.

(1) $$V\;=\;\frac{\pi \cdot d_{K}\cdot A_{N}}{\lambda }$$

For V>2.405, the quantity *N* of the propagating modes can be approximated for step index fibers by Equation (2).

(2) $$N\;=\;\frac{V^{2}}{2}$$

From this equation, the core diameter for single-mode fibers can be derived to $d_{K}\;=\;2.43\;\mu$m for $\lambda_{dye}\;=\;413\;\mu$m (which is the peak wavelength of the 3-vinylayapin dye and $A_{N}\;=\;0.13$ (numerical aperture for *poly*(CHMA-*co*-MMA) with a ratio of 23.3 mol% CHMA and 76.7 mol% MMA, which is used a core material). Core diameters in this range are hard to achieve, even with drawing the whole fiber down to small diameters of 100μm [13,14], which are untypically for polymer fibers. Most of the papers that describe the fabrication of “single-mode polymer optical fibers” do not reach these diameters but stay in the range of 30μm [10], or use other techniques as injection molding [15], microstructuring [16], or two-step processes [17]. Single-mode polymer optical fibers also used to be available commercially by Paradigm Optics Inc. (Vancouver, WA, USA).

**Figure 8:**
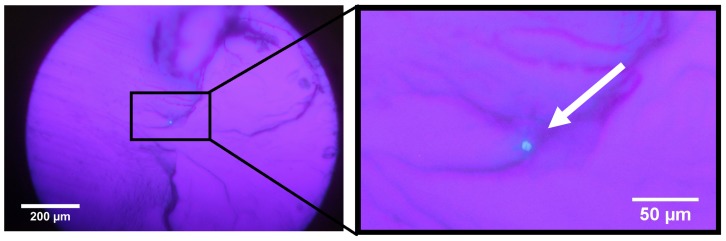
Unpolished Endface of active few-mode fiber under UV illumination at 365 nm. The fiber core (marked with arrow) is doped with 3-vinylayapin **2**. The core diameter is 8.8 microns.

Unlike the fibers named above, the fibers fabricated in this study have large outer diameters as 800μm and small core diameters down to 8.8μm (Figure 8). Using Equation (2), the propagating modes can be calculated to N≈38.

Nevertheless, the fabrication process of the few-mode fiber is still under development. As in other papers that use the string technique, a big issue is that the core monomer mixture is solving the cladding material. The solving effect is kept relatively small by the small core cavity diameters and therefore the smaller interaction area. On the other hand, the small core cavities do not allow a strong prepolymerization of the core material since it becomes harder to fill the higher viscosity gets. Additionally, the small cavity diameters cause strong capillary effects. There is almost no possibility to remove air bubbles that might get stuck in the cavity during the filling process. This also causes a problem as the volume of the monomers shrink up to 15% during the polymerization, which also causes voids inside the fiber core which collapse during the fiber drawing process. In addition, forces occur due to the shrinking process. These forces can be strong enough to bend the strings if they are not tighten thoroughly. Although the preforms were made by hand under lab conditions, the fiber core in the presented fiber is centered relatively well. Nevertheless, the production parameters still get enhanced to increase the reproducibility of that cladding core diameter ratio few mode fibers and the alignment of the core.

## 4. Conclusions

In this study, a analytical method is presented that can give proof if dyes completely polymerize in the polymer chains. From that knowledge, the authors are able to improve the homogeneity of the dye distribution in preforms for optical fibers, which have been fabricated using a free radical bulk polymerization. Furthermore, dye-doped few-mode fibers with an core-cladding ration of 8.8 μm to approximately 800 μm were drawn.

## Figures and Tables

**Table 1:** Process parameters of the studies samples.

Preform Identifier	Doping	Used Process
1-A	vinylayapin	oven
1-B	vinylayapin	orbital shaker
2-A	3-vinylayapin	oven
2-B	3-vinylayapin	orbital shaker

## Abbreviations

The following abbreviations are used in this manuscript:

CHMA	cyclohexyl methacylate
DCM	dichloromethane
FFSC	fluorescent fiber solar concentrator
HPLC	high-performance liquid chromatography
MMA	methyl methacylate
PET	polyethylene terephthalate
PL	photoluminescence
PMMA	*poly*(methyl methacrylate)
POF	polymer optical fiber
PTFE	polytetrafluoroethylene
SEC	size exclusion chromatography
SM	single mode
UV/VIS	ultraviolet-visible
